# What Are the Best Biocompatible Materials for Extracorporeal Membrane Oxygenation

**DOI:** 10.3390/jfb16060226

**Published:** 2025-06-19

**Authors:** Junya Hagiwara, Jeffrey D. DellaVolpe, Yuichi Matsuzaki

**Affiliations:** 1 Institute for Extracorporeal Life Support, San Antonio, TX 78229, USA; junya.hagiwara@ecmo-institute.com (J.H.); jeff.dellavolpe@gmail.com (J.D.D.); 2 Texas IPS, San Antonio, TX 78229, USA; 3 Methodist Heart and Lung Institute Vascular Center, 4410 Medical Dr Ste 420, San Antonio, TX 78229, USA

**Keywords:** biocompatible material, ECMO, hemocompatibility, surface modification, coating technology

## Abstract

Extracorporeal membrane oxygenation (ECMO) is a crucial life support therapy for patients with severe cardiac and respiratory failure. However, the complications associated with venoarterial ECMO (VA-ECMO), including thrombus formation, bleeding, and hemolysis, remain significant challenges that impact patient outcomes and healthcare costs. These complications primarily arise from blood–material interactions within the ECMO circuit, necessitating the development of biocompatible materials to optimize hemocompatibility. This review provides an updated overview of the latest advancements in VA-ECMO materials, focusing on cannula, oxygenators, and centrifugal pumps. Various surface modifications, such as heparin coatings, nitric oxide-releasing polymers, phosphorylcholine (PC)-based coatings, and emerging omniphobic surfaces, have been explored to mitigate thrombosis and bleeding risks. Additionally, novel oxygenator membrane technologies, including zwitterionic polymers and endothelial-mimicking coatings, offer promising strategies to enhance biocompatibility and reduce inflammatory responses. In centrifugal pumps, magnetic levitation systems and hybrid polymer-composite impellers have been introduced to minimize shear stress and thrombogenicity. Despite these advancements, no single material has fully addressed all complications, and further research is needed to refine surface engineering strategies. This review highlights the current progress in ECMO biomaterials and discusses future directions in developing more effective and durable solutions to improve patient safety and clinical outcomes.

## 1. Introduction

Extracorporeal membrane oxygenation (ECMO) is an established therapeutic modality for patients with severe cardiac and/or respiratory failure unresponsive to conventional management. It is employed in both pediatric and adult populations to provide temporary cardiopulmonary support, either as a bridge to recovery or transplantation [1]. In ECMO, blood is drained from the patient via large-bore cannulas inserted into veins or arteries, depending on the ECMO mode. A centrifugal pump propels the blood through a membrane oxygenator, where oxygen is added and carbon dioxide is removed. The oxygenated blood is then returned to the patient’s circulation through another cannula. The ECMO circuit typically includes three key components: cannula, centrifugal pump, and membrane oxygenator. The duration of ECMO support varies depending on the clinical condition and may range from several days to weeks, or, in some cases, even months.

**Figure 1:**
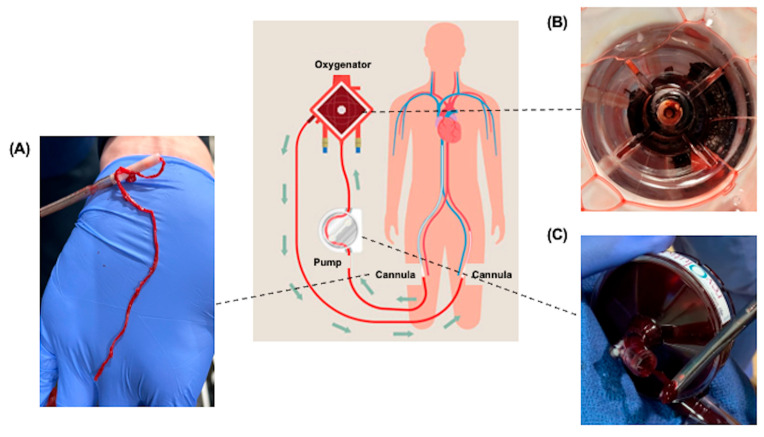
Thrombosis observed in ECMO circuit. (**A**) Thrombus in the cannula, (**B**) thrombus in the oxygenator, (**C**) thrombus in the pump.

However, as illustrated in Figure 1, one of the most significant challenges of ECMO is maintaining hemostasis while simultaneously preventing complications such as thrombus formation and bleeding. In addition, mechanical stress from the ECMO circuit can lead to hemolysis. Thrombus formation can also contribute to hemolysis, making it another critical concern [2,3]. Bleeding is the most frequently observed complication during ECMO, with reported incidence rates ranging from 20–30% [4]. Major thrombotic events occur in approximately 8% of cases, potentially reflecting variability in anticoagulation protocols among institutions [5]. Bleeding may arise at various sites, including surgical wounds, cannulation sites, and intrathoracic, intra-abdominal, retroperitoneal, pulmonary, or intracranial locations. Notably, the incidence of intracerebral hemorrhage has been reported as 3.6% in patients receiving venovenous (VV)-ECMO and 1.5% in those receiving venoarterial (VA)-ECMO [6,7]. A previous study identified predictors of mortality in patients undergoing ECMO, and multivariable analysis demonstrated that hemorrhage was significantly associated with increased mortality [8]. Especially, in VA-ECMO, thrombus formation has been reported in the oxygenator in 4–16% of cases and in the cannula in 29–41% of cases [9,10,11].

Complications increase costs significantly: The median cost of an ECMO hospitalization without complications has been reported to be approximately USD 53,470. The occurrence of a single complication increases this cost to USD 97,560, while the presence of two complications raises it to USD 139,035 [12].

These complications arise from the interaction between blood components and the artificial surface of the ECMO circuit [13]. To reduce the complications of ECMO, biocompatible materials play a crucial role in mitigating these risks by improving hemocompatibility, reducing inflammatory responses, and enhancing the overall safety of ECMO therapy. When considering each component of the ECMO circuit, centrifugal pump failure, although rare in modern ECMO systems (reported in less than 2%), remains one of the most life-threatening complications due to its potential to cause immediate cessation of circulatory support [14]. In contrast, the oxygenator is the most failure-prone component, with circuit or oxygenator exchange required in approximately 20–30% of cases, primarily due to thrombosis and gas exchange dysfunction [15]. Therefore, improving the durability and performance of the oxygenator is a critical priority in clinical practice.

In addition to their hemocompatibility and antithrombotic properties, the clinical application of surface coatings in ECMO circuits is also influenced by regulatory approval processes. Regulatory authorities, such as the U.S. Food and Drug Administration (FDA), the European Medicines Agency (EMA), and the Pharmaceuticals and Medical Devices Agency (PMDA) in Japan, play a key role in evaluating the safety and efficacy of these materials. Nevertheless, improving the biocompatibility of ECMO components has the potential to reduce complications, and thus represents a meaningful effort toward safer and more effective extracorporeal support.

We conducted a narrative review focusing on biocompatible coatings and materials specifically developed for ECMO components, with particular attention to their impact on thrombosis, bleeding, and hemolysis.

## 2. Cannula

Artificial surfaces of ECMO circuits inherently activate coagulation and inflammatory pathways leading to thrombus formation, increased bleeding risk due to anticoagulation therapy, and mechanical damage to red blood cells [16,17,18]. To address these issues, various types of biocompatible coatings have been developed (summarized in Table 1).

### 2.1. Heparin-Coated Surfaces

Heparin-coated surfaces with a colloidal graphite layer prevented clot formation for up to 14 days [19]. Heparin-coated surfaces have been shown to reduce blood transfusion requirements, duration of mechanical ventilation, ICU length of stay, and overall hospital stay [20,21]. However, heparin-coated surfaces do not eliminate the need for systemic anticoagulation during ECMO, so heparin-related complications persist. Though rare, heparin-induced thrombocytopenia (HIT) is a serious complication [22].

### 2.2. Poly-2-Methoxyethylacrylate (PMEA)-Coated Circuits

PMEA is an amphiphilic material characterized by a polyethylene-derived hydrophobic main chain and a side chain with moderate hydrophilic properties [23]. Several studies have demonstrated its potential to reduce platelet activation and minimize surface-mediated protein interactions [23,24,25]. In a swine model, cardiopulmonary bypass (CPB) was associated with reduced complement activation. Additionally, clinical research involving pediatric cardiac surgery patients indicated a suppression of coagulation cascade activation and systemic inflammation [25].

Compared to heparin-coated circuits, PMEA-coated circuits may have the benefit of limiting fibrinogen adsorption and reducing the requirement for intra-procedural platelet supplementation [24]. Nonetheless, several reports have noted an increased occurrence of leukopenia following the procedure and a possible link to systemic inflammatory response syndrome (SIRS) [26]. In addition, no significant differences in platelet aggregation have been observed, and the ability of PMEA coatings to inhibit complement activation may be inferior to that of heparin coatings [25,27,28].

### 2.3. Phosphorylcholine (PC)-Based Coatings

PC is a zwitterionic component commonly found in the outer leaflet of the cell membrane, contributing to its hydrophilic nature. In contrast, the inner leaflet predominantly contains negatively charged phospholipids. While anionic phospholipids are associated with pro-thrombotic activity, PC-containing phospholipids exhibit thromboresistant properties [29,30]. Additionally, PC-coated surfaces mimic the electroneutral characteristics of the endothelial glycocalyx, thereby minimizing electrostatic interactions with plasma proteins and consequently reducing platelet consumption and fibrinogen adsorption [31]. Several studies have associated PC-coated circuits with reductions in postoperative bleeding, transfusion requirements, total heparin dosage, and the incidence of severe complications [32,33]. However, it has also been reported that heparin-coated circuits exhibited fewer thrombotic complications compared to PC-coated circuits, suggesting that the choice of surface coating should be tailored based on clinical context and patient-specific factors [34].

### 2.4. Nitric Oxide (NO)-Releasing Biomimetic Surfaces

**Figure 2:**
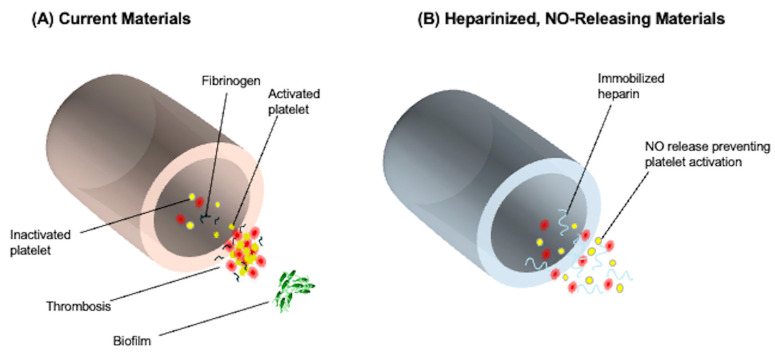
The effect of NO releasing surfaces on blood clot formation in ECMO circuits. (**A**) Standard ECMO circuit materials activate platelets, leading to blood clot formation inside the circuit. Fibrin buildup further increases the risk of blockages, requiring systemic anticoagulation to maintain blood flow. (**B**) NO-releasing biomimetic surfaces prevent platelet activation and clotting, reducing the need for systemic anticoagulation. The addition of immobilized heparin further inhibits fibrin deposition, minimizing the risk of circuit obstruction. NO release also prevents biofilm formation, lowering infection risk and improving overall ECMO biocompatibility. This figure was modified based on the concept described in [38].

NO is an endogenous vasodilator released by endothelial cells, along with prostacyclin, and possesses both direct and indirect anti-platelet effects that inhibit platelet activation and aggregation. The application of NO in ECMO circuits has been widely investigated, particularly for its antithrombotic potential when integrated into polymer materials. Prior studies have summarized strategies utilizing NO to reduce thrombus formation within the circuit [35]. Two principal approaches have been identified: one involves delivering NO into the sweep gas of the membrane oxygenator, while the other enables localized NO release by embedding it within circuit polymers, thereby minimizing systemic exposure. Some reports have demonstrated that NO compounds exhibit excellent platelet inhibition and maintain hemostasis while also leading to fibrinogen consumption [36,37]. As illustrated in Figure 2, recent approaches that embed NO within the polymer matrix of ECMO circuit components have shown that controlled NO release at the surface in contact with circulating blood can reduce platelet activation and thrombus formation, minimize platelet loss, and preserve normal hemostasis, potentially eliminating the need for systemic anticoagulation [36].

### 2.5. Fluid-Repellent Surfaces (Omniphobic)

The design of microstructured coatings that repel liquids by maintaining an air layer at the interface is inspired by natural non-wettable surfaces, such as lotus leaves [39]. Despite their potential, challenges including vulnerability to physical damage and high production costs have limited their practical applications. To address these limitations, surfaces with infused lubricating layers have been designed, demonstrating enhanced liquid and ice repellency, mechanical durability, and optical transparency [39,40]. Building on this concept, bioinspired omniphobic coatings have been developed for medical applications. For instance, modifications of slippery liquid-infused porous surfaces (SLIPS) have been adapted to create anti-thrombogenic coatings for medical-grade materials, effectively preventing fibrin attachment, reducing platelet adhesion and activation, and suppressing biofilm formation [41,42]. In vivo studies have further demonstrated that such coatings can maintain catheter patency without the need for systemic anticoagulation. More recently, chemical vapor deposition (CVD) of fluorine-based organosilanes has been explored as an efficient and non-invasive method for producing omniphobic coatings on catheters. Compared to conventional liquid phase deposition (LPD) techniques, CVD has shown superior reproducibility, reduced disruption of the catheter’s polymeric surface, and enhanced antithrombotic properties. Given these advancements, such surface modifications hold significant potential for improving the biocompatibility and durability of extracorporeal and intracorporeal medical devices [40,43].

**Table 1:** Surface coating technologies for ECMO cannula.

	Mechanism	Metrics	Regulatory Consideration
* Heparin-coated surfaces (Standard globally)	Binds AT to inhibit clotting factors, reducing thrombosis, platelet activation, and complement activation.	Prevent clot formation. Reduce blood transfusion needs. Decrease MV/ICU/hospital duration.	Does not eliminate need for systemic anticoagulation. Risk of HIT.
* PMEA-coated circuits (Asia, Europe, etc.)	Forms a hydrophilic barrier that limits protein adsorption and platelet activation, indirectly suppressing coagulation and inflammation.	Reduce platelet adhesion and aggregation. Decrease fibrinogen adsorption. Attenuate inflammatory response. Reduce need for platelet infusions.	Potential for post-procedural leukopenia. Possible association with SIRS. Compared to heparin coatings, their ability to suppress complement activation may be limited.
* PC-based coatings (Europe, etc.)	Mimics the electroneutral endothelial surface through zwitterionic phospholipids, reducing protein adsorption, platelet activation, and fibrinogen binding.	Mimic endothelial glycocalyx. Reduce fibrinogen adsorption. Avoid HIT risk. Reduce post-operative bleeding and intraoperative thrombosis formation.	Limited efficacy against RBC-dense thrombi under high shear. Mechanical trapping persists in oxygenator meshes.
NO biomimetic surfaces (Experimental)	Releases NO at the blood-contacting surface to inhibit platelet activation and thrombus formation while preserving normal hemostasis.	Prevent thrombosis/platelet activation. Maintain hemostasis. Eliminate need for systemic heparinization.	High temperatures leads to the degradation of NO-releasing capability. Making standard production difficult. Finite NO reservoir depletes after about 4 weeks. Can cause fibrinogen consumption.
Fluid-repellent surfaces (omniphobic) (Experimental)	Uses bioinspired slippery or omniphobic surfaces to repel blood, prevent fibrin and platelet adhesion, and suppress biofilm formation.	Prevent fibrin deposition. Reduce platelet adhesion/activation. Inhibit the development of biofilms. Maintain catheter patency without systemic anticoagulation.	Vulnerability to physical damage. High production costs. Challenges in practical applications despite promising results.
Heparin plus NO-releasing surfaces (Experimental)	Combines multiple surface modification techniques to simultaneously inhibit platelet activation, reduce thrombogenicity, and improve antibacterial performance.	Prevent platelet adhesion and activation. Reduce fibrin formation; enhance hemocompatibility. Potential reduction of systemic anticoagulation requirement.	Challenges in uniform and durable coating application. Complex regulatory assessment due to multiple functional components. Stability and effectiveness under clinical flow conditions require further validation.

* Rows with asterisk indicate coating technologies that are currently applied in clinical practice. Abbreviation: AT, antithrombin; MV, mechanical ventilation; ICU, intensive care unit; HIT, heparin-induced thrombocytopenia; SIRS, systemic inflammatory response syndrome.

## 3. Future Directions in ECMO Cannulas

Recently, efforts to enhance the biocompatibility of ECMO cannulas have focused on advanced materials, such as zwitterionic polymer coatings [44,45], tethered-liquid perfluorocarbon (TLP) coatings [46,47], hydrogel-based surfaces [48,49], and biomimetic endothelial coatings [50,51]. Additionally, emerging technologies, including nanocoatings that combine antimicrobial properties with coagulation factor XII (FXII)-targeted antithrombotic mechanisms [52,53] and smart cannulas with real-time thrombus detection [54], show promise but require further refinement to optimize clinical safety and effectiveness. These advancements may contribute to reducing thrombogenicity and help create a safer ECMO environment by potentially lowering the required intensity of anticoagulation.

## 4. Oxygenator

The oxygenator is a major thrombogenic component within the ECMO circuit due to its large surface area and the presence of air–blood interfaces. The blood-contacting surface area of oxygenators typically ranges from 1.8 to 2.5 m^2^ in adults and from 0.3 to 0.8 m^2^ in pediatric cases, with corresponding priming volumes of 200–250 mL and 30–100 mL, respectively [55,56]. This extensive contact area promotes activation of inflammatory pathways and coagulation cascades upon exposure to non-endothelialized materials. Compared to other extracorporeal devices, ECMO presents a significantly greater blood–material interface, amplifying the risk of thrombus formation and hemolysis. To mitigate these effects, strategies such as systemic anticoagulation and surface modification of circuit materials are employed, although optimizing biocompatibility remains a substantial clinical challenge.

Hemorrhagic complications are observed in 20–30% of adult ECMO patients, while thrombotic events occur in around 10% [4,57]. Based on the cell-based coagulation model, two primary approaches are considered effective for minimizing thrombus formation: (1) suppressing fibrin adhesion to the material surface and (2) inhibiting thrombin activation [58]. Membranes used in oxygenators must possess key properties such as excellent biocompatibility and hydrophobicity. Among various design strategies, surface engineering of membrane materials remains a major focus in ECMO development (as shown in Table 2). Researchers have explored a wide array of techniques, which can be broadly categorized as (1) modifying surface physicochemical characteristics to reduce reactivity with blood components and (2) constructing biomimetic interfaces to improve hemocompatibility [58,59,60].

### 4.1. Physicochemical Surface Engineering

Modifying the physicochemical characteristics of membrane surfaces can help reduce their interaction with blood components. Various strategies have been employed to alter ECMO membrane surfaces, including chemical grafting, physical adsorption, and ion implantation.

#### 4.1.1. Surface Chemical Modification

Surface chemical modification is a common approach to improving the hemocompatibility of ECMO membranes. Initially, hydrophobic modifications, such as poly (L-lactide) (PLLA) and poly (2-methoxyethyl acrylate) (PMEA) [61], were used to reduce blood leakage, but they led to increased protein adhesion. To address this, hydrophilic modifications using polymers like polyethylene oxide (PEO) were introduced, as they create a hydration layer that repels proteins and enhances biocompatibility. PEO, known for its oxidation stability, has been successfully grafted onto stable surfaces like silicon, glass, and gold [62,63]. Another strategy involves zwitterionic polymer coatings, where polymers with both positive and negative charges, such as carboxyl betaine and betaine sulfate, effectively resist bacterial adhesion, platelet adhesion, and protein adsorption. Researchers have applied these polymers to various membranes, including poly (CABA) on polypropylene membranes using plasma treatment and UV grafting, and sulfobetaine-modified polysulfone (PSU) membranes, which demonstrate improved antifouling properties [64].

#### 4.1.2. Physical Absorption Modification

Surface chemical modification is a commonly used strategy to enhance the hemocompatibility of ECMO membranes. Early approaches employed hydrophobic polymers to prevent blood leakage through the membrane. However, these materials often increased the risk of protein adsorption and platelet activation. To address this limitation, hydrophilic polymers were applied to the membrane surface to form a hydration layer that minimizes direct contact between blood components and the material [65,66]. This water-rich barrier helps prevent protein binding and reduces thrombus formation. Examples include coatings based on polyether compounds, such as those used to improve oxygenator performance in long-term ECMO support. More recently, surface designs that imitate the outer structure of biological cell membranes have gained attention. These biomimetic coatings incorporate both hydrophilic and hydrophobic regions to replicate the natural phospholipid bilayer. Such surfaces are known to resist protein fouling, platelet adhesion, and bacterial colonization. These properties contribute to improved blood compatibility and may help reduce complications during prolonged extracorporeal circulation [67].

#### 4.1.3. Plasma Deposition

Plasma chemical vapor deposition (PCVD) is a surface engineering technique that uses low-temperature plasma to activate reactive gases, promoting chemical reactions on material surfaces to form thin solid films [68]. The process involves ionizing the gas source using a high-frequency or direct current electric field, allowing chemical bonds to break and reform, which introduces functional groups without excessive heat. PCVD also enables the introduction of amine groups onto poly(4-methyl-1-pentene) PMP fibers, which allows the subsequent grafting of amphoteric polymers such as sulfonate betaine, resulting in an 80–95% reduction in platelet adhesion compared to unmodified PMP [29]. Despite its effectiveness, PCVD consumes more energy than other surface modification methods.

#### 4.1.4. Self-Assembly Technology

The layer-by-layer (LbL) technique is a commonly used method to create thin protective coatings on medical device surfaces by stacking multiple layers through mild chemical interactions [69]. However, this approach can be challenging when using zwitterionic polymer coatings, which have both positive and negative charges, because they do not easily form stable layers. To address this, researchers have developed more durable coatings by chemically linking the layers together. One example involves using a molecule called 3,4-dihydroxyphenylalanine (DOPA), which naturally forms a strong adhesive coating known as polydopamine [70,71]. By combining an LbL assembly with advanced chemical techniques such as “click chemistry,” scientists have successfully applied stable coatings onto oxygenator membranes, which help reduce protein and platelet adhesion and delay blood clotting. For instance, a membrane coated with paclitaxel and chitosan layers showed improved blood compatibility, reducing the attachment of albumin and fibrinogen by 60–70% and platelet adhesion by 94% [72,73].

#### 4.1.5. Surface Structure Design

Recent research has suggested that modifying the surface of oxygenator membranes with gradual chemical changes can help reduce clot formation and improve compatibility with blood [74,75]. Several techniques—such as adjusting surface chemistry, structure, temperature, or energy exposure—have been studied to create these effects without requiring complex procedures. One example is a laser-based method that alters the surface of silicone materials like polydimethylsiloxane (PDMS) in a single step, making them less likely to attract proteins or cells, improving blood flow, and lowering the risk of red blood cell damage [76]. In some cases, an additional coating with fatty acids can make the surface extremely water-repellent and capable of cleaning itself, which helps prevent blood components from sticking [77]. Another method uses a special oil-based solution to create surfaces with gradual changes in how they interact with fluids [78]. However, keeping these water-repellent properties stable when the surface is in contact with blood or other fluids remains difficult, which limits their use in real-world clinical settings.

### 4.2. Biomimetic Interface Design

Endothelial cells (ECs) form a monolayer along the luminal surface of blood vessels, functioning as a critical barrier between circulating blood and the vascular wall [79]. They are actively involved in metabolic exchange, control of vascular tone, and the regulation of procoagulant and anticoagulant pathways. The endothelial membrane naturally expresses anticoagulant factors such as prostaglandin and heparin, which contribute to thrombus prevention. In addition to physically covering the vessel wall, ECs secrete anti-inflammatory mediators that further suppress coagulation and support vascular homeostasis.

#### 4.2.1. Surface Endothelialization

**Figure 3:**
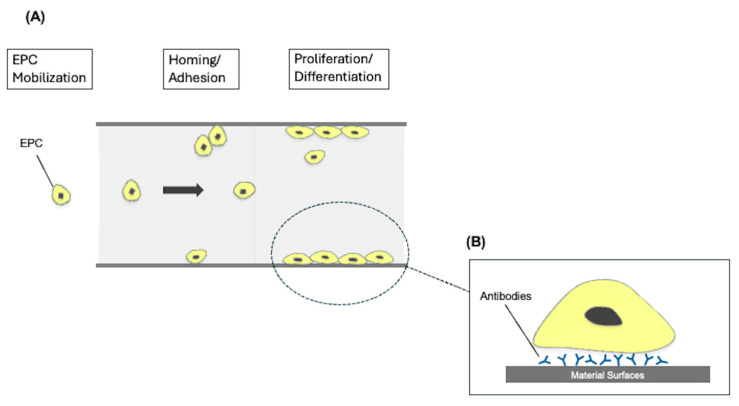
A schematic illustration of the biomimetic endothelialization. (**A**) Endothelial progenitor cells (EPCs) are released from the bone marrow into the peripheral circulation in response to vascular injury or ischemic stimuli. (**B**) Circulating EPCs are selectively captured at the implantation site via surface-immobilized antibodies (e.g., anti-CD34, anti-VEGFR2) that recognize specific receptors on EPCs. These interactions facilitate EPC adhesion to the biomaterial surface, where they proliferate and differentiate into mature endothelial cells, forming a functional endothelial lining that promotes hemocompatibility and vascular healing. This figure was modified based on the concept described in [82].

Endothelialization of anticoagulant material surfaces helps reduce thrombus formation and platelet activation, improving blood compatibility by expressing anti-thrombosis and anti-inflammatory molecules [80,81]. This can be achieved through two approaches: in vitro pre-endothelialization, where autologous endothelial cells are implanted onto synthetic vascular grafts before use, and in vivo self-endothelialization, where endothelial progenitor cells (EPCs) differentiate into mature endothelial cells to support vascular repair (Figure 3) [82]. While endothelialization shows promise for ECMO membranes, its complex structure and technical challenges make it difficult to implement effectively, and research in this area remains underdeveloped. Recent studies have demonstrated the feasibility of establishing an endothelial monolayer on PMP-based gas-exchange membranes of ECMO devices using titanium dioxide coatings [83].

#### 4.2.2. Graft Phosphorylcholine (PC)

Since it is technically difficult to create a complete layer of endothelial cells on artificial surfaces, coating materials with components that mimic the natural blood vessel lining have become a promising approach. One such component is PC, a water-attracting molecule found in cell membranes. When applied to polymer surfaces like PDMS, PC coatings can reduce platelet adhesion and decrease the attachment of proteins—both of which are key to preventing clot formation [84,85]. Recent developments include synthetic polymers that combine PC with similar molecules, further improving biocompatibility. Notably, coating ECMO oxygenator membranes made from PMP with a PC layer has been shown to significantly improve their compatibility with blood [29].

#### 4.2.3. Graft Protein

The use of protein coatings, derived from either natural or synthetic sources, has been explored to reduce thrombus formation on ECMO circuit surfaces by limiting fibrin deposition and platelet activation. Albumin was one of the earliest proteins applied for surface modification [86]. When immobilized onto hydrophobic materials, albumin enhances surface hydrophilicity and competes with plasma proteins, thereby contributing to improved hemocompatibility. In addition to albumin, other proteins and enzymes have been investigated to enhance hemocompatibility. Thrombomodulin, an endothelial cell surface protein, has been covalently immobilized on artificial surfaces to inhibit coagulation and complement activation [87]. Furthermore, antibody-based strategies have been explored. Anti-CD34 antibodies immobilized on biomaterial surfaces can capture circulating endothelial progenitor cells, promoting endothelialization and improving thromboresistance [88].

#### 4.2.4. Graft Tissue Plasminogen Activator (tPA)

tPA is a fibrinolytic enzyme that promotes the breakdown of fibrin clots. Surface coatings with immobilized tPA have been developed to provide localized thrombolytic activity on ECMO circuit components. When applied to PDMS or polymer brush surfaces, tPA coatings help delay clot formation and promote clot lysis in response to fibrin exposure [89,90]. These coatings mimic the endothelium’s natural fibrinolytic function and may reduce the need for systemic anticoagulation. While still experimental, tPA-based strategies show potential to improve hemocompatibility in oxygenators under flow conditions.

**Table 2:** Biocompatibility enhancement technologies for ECMO oxygenators.

	Mechanism	Metrics	Regulatory Consideration
Surface chemical modification	Introduces hydrophilic or zwitterionic polymer layers. These layers form hydration barriers that reduce protein adsorption and platelet adhesion.	Improves hemocompatibility. Reduces thrombosis, bacterial adhesion, and inflammatory response.	Complex processing, not yet standard for all ECMO oxygenators.
Physical absorption modification	Applies anticoagulant or hydrophilic compounds via weak interactions to reduce fouling.	Simple to implement. Reduces protein and platelet adhesion.	Weak adhesion and reversibility limit durability. Requires surface-stabilizing additives.
Plasma deposition	Uses plasma to activate reactive gases and form chemical bonds with membrane surfaces. Enables polymer grafting and functional group integration.	Reduces platelet adhesion. Allows functional tuning of surfaces for blood compatibility.	High energy consumption and specialized equipment are required. Large-scale production remains challenging.
Self-assembly technology	Constructs stable multilayer coatings using mild chemical interactions. Utilizes molecules that promote adhesion and layering.	Reduces protein adhesion and platelet adhesion. Improves blood compatibility.	Stabilization of zwitterionic coatings is difficult. Requires advanced chemical techniques.
Surface structure design	Alters membrane surfaces with lasers or chemical gradients to create hydrophobic or omniphobic features that repel blood.	Reduces thrombosis and hemolysis. Prevents protein and cell adhesion.	Superhydrophobic properties degrade in wet environments. Clinical stability over time is limited.
Surface endothelialization	Promotes attachment and growth of endothelial or progenitor cells to mimic the vascular lining.	Reduces platelet activation and clot formation. Improves overall blood compatibility.	Technically complex. Still in the research phase for ECMO applications.
Graft phosphorylcholine (PC)	Mimics the phospholipid layer of natural cell membranes to reduce platelet and protein adhesion.	Enhances membrane biocompatibility. Reduces thrombosis and bleeding risk.	Limited availability for mass production. Advanced synthesis methods required.
Graft protein	Immobilizes functional proteins on the membrane surface to block coagulation pathways and reduce adhesion.	Improves hemocompatibility. Suppresses fibrin and platelet activation.	Protein coatings may degrade over time. Long-term effectiveness remains a concern.
Graft tissue plasminogen activator (tPA)	Immobilizes tPA to provide local fibrinolytic activity that promotes clot breakdown in response to fibrin.	Delays clot formation. Mimics natural endothelial fibrinolysis.	Still experimental. Stability and uniformity under clinical conditions need further validation.

## 5. Future Directions in ECMO Oxygenators

A key consideration for the long-term application of ECMO oxygenators is the improvement of membrane material properties, with oxygen and carbon dioxide exchange efficiency as well as flow characteristics serving as critical performance indicators. Future strategies may include multi-layer surface modifications that combine hydrophilic polymers (e.g., polyethylene glycol) with anticoagulant coatings such as heparin or nitric oxide-releasing compounds to reduce protein adsorption and thrombus formation while maintaining gas permeability [58]. Furthermore, the advancement of nano-structured or porous coatings—such as silicone-based or fluoropolymer materials—can enhance gas transfer rates by increasing surface area and reducing diffusion resistance [91,92]. Simultaneously, integrating real-time biosensors, such as ultrasonic sensors that detect thrombi by changes in acoustic signals and bioimpedance sensors that capture early shifts in electrical resistance, may allow for earlier intervention and device replacement, improving ECMO safety and efficacy [54,93].

## 6. Centrifugal Pump

The centrifugal pump is a critical component of ECMO systems, yet its operation introduces significant risks of thrombosis due to the interplay of fluid dynamics, mechanical stress, and biological responses. Thrombosis in these pumps arises from complex mechanisms involving shear-induced platelet activation, flow stagnation, recirculation zones, and operational mismatches between pump design and clinical use [13,94,95]. Regarding surface engineering, its principles are similar to those of cannulas and oxygenators; therefore, we focus on recent material innovations (as shown in Table 3).

### 6.1. Magnetic Levitation Systems

Magnetic levitation systems (MagLev) in ECMO refer to advanced centrifugal pump technology where the impeller is suspended and rotated without mechanical contact, using magnetic forces [96]. Magnetic levitation pumps minimize blood damage and clot formation due to contact-free impeller rotation, eliminating friction and wear, optimized flow dynamics with reduced blood stagnation areas, and lower shear stress on blood cells [96,97]. These pumps demonstrate improved longevity and efficiency, featuring precise rotor control with 32,000 adjustments per second and automatic compensation for external movements. This technology has the potential to extend ECMO support without frequent equipment changes, thereby enhancing durability and performance [98]. This innovation has demonstrated reduced hemolysis and enhanced hemocompatibility compared to conventional pump systems [97,98,99]. Future iterations may integrate adaptive levitation algorithms that dynamically adjust impeller positioning based on real-time hematocrit and viscosity measurements.

### 6.2. Hybrid Polymer-Composite Impellers

Hybrid polymer-composite impellers integrate high-performance polymers such as polyether ether ketone (PEEK) and ultra-high molecular weight polytheylene (UHMWPE) with advanced coatings to enhance both mechanical durability and blood compatibility [100]. These polymers provide high wear resistance, low friction, and excellent fatigue strength, making them well suited for long-term implantation in centrifugal pumps [100]. To further improve hemocompatibility, hydrophilic surface coatings like 2-methacryloyloxyethyl phosphorylcholine (MPC) polymer or hydrogel layers are applied, reducing protein adhesion and minimizing platelet activation [101]. Additionally, drug-eluting coatings incorporating anticoagulants such as bivalirudin enable localized thrombus prevention, lowering the need for systemic anticoagulation and thereby reducing bleeding risks [102]. Nanocomposite polymers infused with heparin-mimicking nanoparticles or graphene oxide are also being explored to provide inherent anticoagulant properties while maintaining structural integrity [100]. Future developments aim to integrate stimuli-responsive coatings that dynamically release antithrombotic agents in response to shear stress or clot formation, ensuring sustained biocompatibility. These advancements in hybrid polymer-composite impellers are expected to significantly improve long-term reliability, hemocompatibility, and safety in next-generation blood pumps.

**Table 3:** Biocompatibility characteristics of ECMO pump types.

	Mechanism	Metrics	Regulatory Consideration
Magnetic Levitation Systems	Impeller is suspended using magnetic forces. Eliminates mechanical contact, friction, and wear. Minimizes shear stress and blood stagnation.	Reduce hemolysis. Improve blood compatibility/long-term durability/reliability. Enhance flow stability/efficiency.	Higher cost. Complex system/ maintenance. Larger size. Limited portability. Potential for magnetic interference.
Hybrid Polymer -Composite Impellers	Use of durable polymers with surface coatings. Coated with hydrophilic polymers or drug-eluting agents. Nanocomposite materials with inherent anticoagulant features.	Enhance mechanical durability. Improve hemocompatibility. Inherent anticoagulant properties. Improve safety and long-term reliability.	Potential for material degradation. Limited clinical data.

## 7. Conclusions

ECMO is essential for patients with severe cardiac and respiratory failure, but complications such as thrombosis, bleeding, and hemolysis remain major challenges. This review has highlighted recent advances in biocompatible materials that have shown promise in mitigating these complications by enhancing hemocompatibility, reducing platelet activation, and improving durability. Innovations like endothelial-mimetic surfaces and nanomaterials may enhance oxygenator performance, while magnetic levitation and composite impellers may reduce hemolysis in pumps. However, effectively integrating these biomaterials into clinical ECMO systems requires a systematic approach. Collaborative efforts between materials science, engineering, and clinical practice are needed to address the challenges of safety, durability, and cost. Furthermore, harmonizing the performance of these advanced materials across different ECMO components is essential to realize their full clinical potential. Therefore, future research should focus not only on developing multifunctional coatings and novel materials but also on creating integrated strategies that bridge laboratory innovation with clinical application, ultimately improving patient outcomes and reducing complications.
